# Population-level factors associated with maternal mortality in the United States, 1997–2012

**DOI:** 10.1186/s12889-018-5935-2

**Published:** 2018-08-13

**Authors:** Daniel B. Nelson, Michelle H. Moniz, Matthew M. Davis

**Affiliations:** 1 Harvard Kennedy School of Government, Cambridge, MA USA; 2 University of Michigan Medical School, 1500 E Medical Center Drive, Ann Arbor, MI 48109 USA; 3 Department of Obstetrics and Gynecology, University of Michigan Medical School, Ann Arbor, MI USA; 4 Academic General Pediatrics and Primary Care, Department of Pediatrics, Ann & Robert H. Lurie Children’s Hospital, Chicago, IL USA; 5 Mary Ann & J. Milburn Smith Child Health Research, Outreach, and Advocacy Center, Ann & Robert H. Lurie Children’s Hospital, Chicago, IL USA; 6 Departments of Pediatrics, Medicine, Medical Social Sciences, and Preventive Medicine, Northwestern University Feinberg School of Medicine, Chicago, IL USA

**Keywords:** Maternal mortality, Health policy, Obesity, Race/ethnicity, Chronic disease

## Abstract

**Background:**

In contrast to peer nations, the United States is experiencing rapid increases in maternal mortality. Trends in individual and population-level demographic factors and health trends may play a role in this change.

**Methods:**

We analyzed state-level maternal mortality for the years 1997–2012 using multilevel mixed-effects regression grouped by state, using publicly available data including whether a state had adopted the 2003 U.S. Standard Certificate of Death, designed to simplify identification of pregnant and recently pregnant decedents. We calculated the proportion of the increase in maternal mortality attributable to specific factors during the study period.

**Results:**

Maternal mortality was associated with higher population prevalence of obesity and high school non-completion among women of childbearing age; these factors explained 31.0% and 5.3% of the attributable increase in maternal mortality during the study period, respectively. Among delivering mothers, prevalence of diabetes (17.0%), attending fewer than 10 prenatal visits (4.9%), and African American race (2.0%) were also associated with higher maternal mortality, as was time-varying state adoption of the 2003 death certificate (31.1%).

**Conclusions:**

Our findings indicate that, in addition to better case ascertainment of maternal deaths, adverse changes in chronic diseases, insufficient healthcare access, and social determinants of health represent identifiable risks for maternal mortality that merit prompt attention in population-directed interventions and health policies.

**Electronic supplementary material:**

The online version of this article (10.1186/s12889-018-5935-2) contains supplementary material, which is available to authorized users.

## Background

Following decades of improvement in maternal health in the United States, the maternal mortality ratio has more than doubled over the last two decades from 9.8 maternal deaths per 100,000 live births in 2000 to 21.5 per 100,000 in 2014 [1–3]. Over the same period, the vast majority of industrialized nations have achieved reductions in maternal mortality [4].

Some of the U.S. increase in maternal mortality is attributable to changes in ascertainment of maternal deaths related to an enhancement in death certificate coding progressively adopted by states since 2003 [3]. Nonetheless, many experts have suggested that one-quarter to as many as one-half of maternal deaths are preventable [5–9]. Many individual-level factors may explain worsening U.S. obstetric outcomes over the last two decades, such as a temporal trends in the number of births to women of advanced maternal age [10], increased prevalence of obesity and other chronic health conditions [11, 12], and an increase in the rate of cesarean sections [8, 13]. Given known racial disparities in maternal mortality [2, 14], factors related to social determinants of health—including insurance status, income, and education—may also help explain current trends.

Population-wide increases in maternal mortality are difficult to attribute solely to trends in individual-level factors, however. Instead, understanding how population-level factors change and relate to maternal mortality over time may provide insights into potentially effective public health and health policy responses. Given that states vary widely in their maternal mortality rates over time, and that states also vary substantially with respect to several population-level factors that may be associated with maternal mortality, we conducted state-level analyses of trends in maternal mortality to provide a lens for potential population-level interventions.

## Methods

We conducted a secondary data analysis of publicly available data to identify factors independently associated with maternal mortality in each state. Our outcome of interest was state-level maternal mortality, defined as all women who died within 42 days of pregnancy termination in which the pregnancy contributed to the cause of death. Our predictors included maternal health characteristics, measures of obstetric morbidity, sociodemographic information, and health care factors.

### Data sources

We obtained maternal mortality counts for each state and the District of Columbia for the years 1995–2014 from the National Vital Statistics System compressed mortality files. All vital statistics data were downloaded via the Centers for Disease Control and Prevention (CDC) WONDER tool (wonder.cdc.gov), with the exception of total prenatal visits, which were obtained directly from NVSS public use live birth files.

Maternal deaths were categorized by the CDC based on ICD-9 codes 630–676 (years 1995–1998) or ICD-10 codes A34, O00-O95, and O98-O99 (years 1999–2013). The changes to ICD coding procedures are known to have led to an increase in the number of maternal deaths coded. Maternal mortality counts from 1995 to 1998 were therefore multiplied by the CDC correction factor to enable historical comparisons between the ICD versions [12, 15]. Given significant year-to-year variability in maternal mortality for many states, including some zero values in low-population states, we generated five-year moving averages for all maternal mortality rates, with the total maternal deaths and live births from 2 years before and 2 years after the named year used in calculation of maternal mortality rates. Thus, our analysis reflects outcomes from 1997 to 2012, because source data included 1995–2014. All maternal mortality rates were calculated as per 100,000 total live births.

Our predictors of maternal mortality were measured in each state in each study year. Information obtained from live birth certificates and aggregated at state level included cesarean section rate, maternal age, maternal race, gestational age at delivery, prenatal care patterns (mean number of visits, gestation age at presentation, proportion of pregnant women with a live birth who attended fewer than 10 prenatal visits), and maternal prevalence of diabetes, pregnancy-related hypertension, and chronic hypertension. Birth certificate data do not differentiate between types of diabetes present in the mother. Data regarding obesity, tobacco use, alcohol consumption, educational attainment, and self-reported health status among women of childbearing age were obtained from the Behavioral Risk Factor Surveillance System (BRFSS). Childbearing age was defined as 18–44 years old, given constraints of BRFSS sampling of adults 18 years and older. Educational status was defined as proportion not having completed high school or General Equivalency Diploma (GED), and self-reported health status was categorized as proportion rating their health status as “fair” or “poor”. Insurance status was obtained from the Annual Social and Economic Supplement of the Current Population Survey (CPS ASEC) and median household income was obtained from the American Community Survey, both administered by the U.S. Census Bureau. To obtain obstetric workforce trends, we calculated the number of obstetricians in active patient care in each state (as number per 100,000 live births), using data from the Area Health Resource Files from the U.S. Health Resources and Services Administration.

In 2003, the U.S. developed and disseminated an updated version of the Standard Certificate of Death [3, 16]. The revised death certificate included a checkbox with questions asking about the pregnancy status of female decedents in order to theoretically increase ascertainment of pregnancy-related death. State governments were given the discretion to adopt this new certificate when they chose to do so. Information on the year in which each state adopted the 2003 Revision of the U.S. Standard Certificate of Death was obtained from the Division of Vital Statistics in the National Center for Health Statistics (Karen Knight, personal communication); i.e., implementation of the revised death certificate occurred at different times across the United States and DC, making a typical point-in-time policy analysis inappropriate. As this change in certificate has been associated with improved ascertainment of maternal mortality events [3], we generated an indicator variable for whether a state had adopted the revised death certificate in a given year, and included that variable in all analyses.

### Analyses

We performed multilevel mixed-effects linear regression analysis for maternal mortality on each candidate population-level predictor variable, specifying grouping by state. The two levels are year (level 1) and state (level 2). We calculated an intraclass correlation coefficient for level 2 to characterize the degree of similarity of observations within states over time. Given the known ascertainment bias related to adoption of the 2003 revised death certificate, all regression analyses included the indicator variable for year in which the revised death certificate was adopted by each state [see Additional file 1 for regression details]. In other words, each initial model to evaluate the association between a candidate predictor variable and maternal mortality also included the year of revised death certificate implementation, calendar year, and state.

Candidate predictor variables associated with maternal mortality at *p* < .05 in initial models were subsequently considered for inclusion in a final model. To avoid overspecification related to multicollinearity, when pairs of candidate predictor variables had pairwise *r* > 0.5, only the variable with the larger *z* score in its initial model was retained as a candidate predictor for the final model. To achieve a parsimonious final model given the modest number of observations, after the multilevel mixed-effects multivariable model was run, variables with associations with maternal mortality at *P* > 0.05 were removed. The most parsimonious multilevel mixed-effects multivariable regression model of maternal mortality was then fit and is presented as the final model.

To estimate the relative magnitude to which the variables retained in the final model are associated with maternal mortality rates, the proportion of the observed increase in maternal mortality attributable to time trends were estimated for each factor. The numerical change in each variable at the state level (1997 vs. 2012) was multiplied by the regression coefficient and divided by the overall change in maternal mortality over the same time period. The state-level values were then averaged to calculate the overall percent attributable for each factor.

All statistical analyses were performed with Stata software (version 14, StataCorp, College Station, TX). This secondary analysis of de-identified, publicly available data was deemed not regulated by the University of Michigan Medical School Institutional Review Board.

## Results

**Fig. 1:**
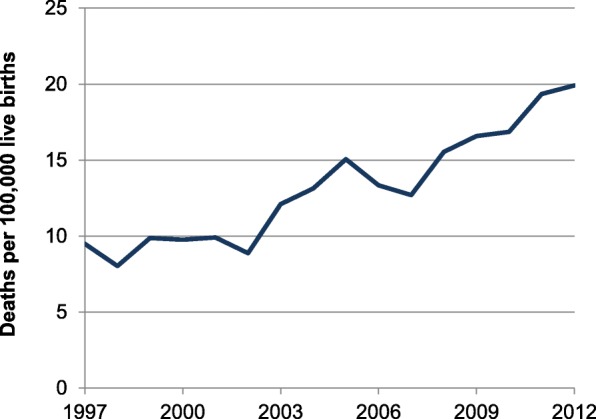
Trends in overall maternal mortality rate – United States, 1997–2012*. *Rates are five-year moving averages including data from the years 1995–2014, for the United States in aggregate

**Fig. 2:**
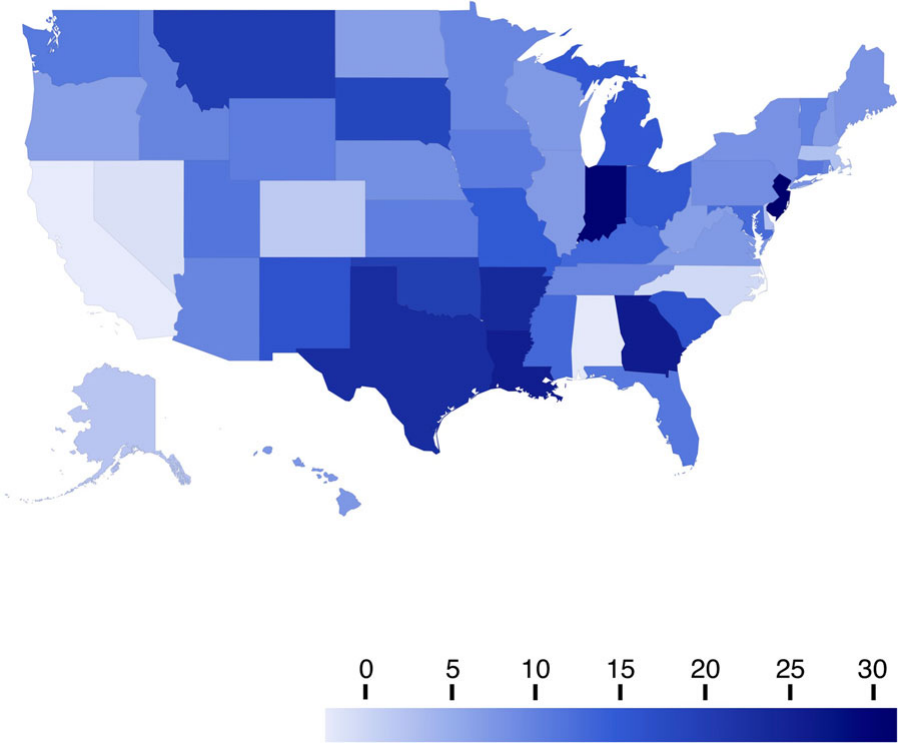
Changes in maternal mortality rates by state – United States, 1997–2012. Rates are presented as maternal deaths per 100,000 live births. Change values were calculated by the authors based on 5-year moving averages for the years 1997 and 2012, based on data from the years 1995–2014

The raw maternal mortality rate in the United States increased from 9.5 deaths per 100,000 live births in 1997 to 19.9 deaths per 100,000 live births in 2012 (Fig. 1). There was significant state-level variation in maternal mortality trends during the study period (Fig. 2).

Results of initial analyses (regressing maternal mortality on each candidate predictor variable and the year of implementation of the revised death certificate indicator, with year and state as covariates) are presented in Table 1. Fifteen distinct population-level variables were associated with maternal mortality at *P* < 0.05 and thus were candidates for the final model. Seven variables were subsequently eliminated because of collinearity with other candidate variables that had stronger associations with maternal mortality based on *z* scores in the initial models. State-level data regarding proportions of births to women over 40 years of age, women with diabetes, women with pregnancy-related hypertension, African American women, and women attending fewer than 10 prenatal visits were ultimately included in the next phase of multilevel mixed-effects multivariable modeling, as were prevalence among women of childbearing age of obesity and smoking, and the proportion not having completed high school or a GED. The intraclass correlation coefficient was 0.41 for this model.

**Table 1:** Factors associate with maternal mortality at the state level, in initial regression analyses – United States, 1997–2012

State-Level Variable	Regression Coefficient	95% CI	*P* value
Proportion of births to women with chronic hypertension	+ 3.82	[2.99–4.65]	<.001
Proportion of births to women over 40 years of age	+ 1.82	[0.90–2.74]	<.001
Proportion of births to women with diabetes	+ 1.24	[0.94–1.53]	<.001
Proportion of births to women with pregnancy-related hypertension	+ 0.98	[0.49–1.47]	<.001
Proportion of women of childbearing age with self-reported health status of “Fair” or “Poor”	+ 0.53	[0.37–0.69]	<.001
Proportion of women of childbearing age with BMI ≥ 30	+ 0.38	[0.30–0.45]	<.001
Proportion of births by cesarean delivery	+ 0.35	[0.27–0.42]	<.001
Proportion of women of childbearing age not having completed high school/GED	+ 0.31	[0.18–0.45]	<.001
Proportion of births to African American women	+ 0.25	[0.19–0.32]	<.001
Median household income, in thousands	+ 0.21	[0.15–0.28]	<.001
Proportion of births to Hispanic women	+ 0.17	[0.08–0.27]	<.001
Uninsurance rate among women of childbearing age	+ 0.14	[0.04–0.24]	.007
Proportion of births to women who attended fewer than 10 prenatal visits	+ 0.12	[0.05–0.19]	.001
Proportion of women of childbearing age reporting alcohol consumption in previous 30 days	+ 0.09	[0.02–0.16]	.01
Proportion of women of childbearing age who are current smokers	−0.18	[− 0.27 – − 0.08]	<.001

All analyses are adjusted for adoption of the 2003 Revision of the U.S. Standard Certificate of Death. All linear regression models were specified as multilevel mixed-effects, with calendar year as level 1 and state as level 2. BMI: Body Mass Index; GED: General Equivalency Diploma

In the most parsimonious final model that included only predictor variables associated with maternal mortality at *P* < 0.05, time trends in obesity, diabetes, high school education, African American race, and fewer than 10 prenatal care visits, along with the revised death certificate indicator variable, were all significantly positively associated with state-level trends in maternal mortality (Table 2). Implementation of the revised death certificate was associated with an estimated increase in maternal mortality of more than 6 deaths per 100,000 births. Meanwhile, a 1% increase in prevalence of obesity among women of childbearing age was associated with a + 0.24 unit increase in maternal mortality rate. A 1% increase in diabetes prevalence among women of childbearing age was associated with a + 0.39 unit change in maternal mortality. High school non-completion, African American race among childbearing women, and attending fewer than 10 prenatal visits were also positively associated with maternal mortality.

**Table 2:** Multilevel mixed-effects multivariable linear regression model of maternal mortality – United States, 1997–2012

Predictive Variable	Coefficient	95% CI	*P* value	Share of change in maternal mortality attributable to factor^a^
Adoption of 2003 Revision of the U.S. Standard Certificate of Death by 2011	+ 6.26	[5.41–7.11]	<.001	31.1%
Proportion of women of childbearing age with BMI ≥ 30	+ 0.25	[0.15–0.34]	<.001	31.0%
Proportion of births to women with diabetes	+ 0.39	[0.04–0.75]	.03	17.0%
Proportion of women of childbearing age not having completed high school/GED	+ 0.17	[0.05–0.30]	.005	5.3%
Proportion of births to women who attended fewer than 10 prenatal visits	+ 0.07	[0.01–0.14]	.03	4.9%
Proportion of births to African American women	+ 0.20	[0.14–0.27]	<.001	2.0%

^a^Percentages sum to 91.3% of the time trend in maternal mortality attributable to the factors retained in the most parsimonious final model. Candidate predictor variables for the multilevel mixed-effects multivariable model that had *P* > 0.05 were removed from the most parsimonious final model shown here. BMI: Body Mass Index; GED: General Equivalency Diploma

The most parsimonious final multi-level model was able to explain 91% of the increase in maternal mortality over the study period. Thirty-one percent of the increase was attributable to the adoption of the revised death certificate. Another 31% was attributable to the proportion of obese women of childbearing age, followed by proportion of births to women with diabetes (17%), women of childbearing age not having completed high school (5.3%), births to women attending fewer than 10 prenatal visits (4.9%), and births to African American women (2%).

## Discussion

Our analysis examines state-level maternal mortality rates and their associations with many potential risk factors. Factors such as advancing maternal age, better ascertainment of maternal deaths, increased prevalence of obesity and chronic health conditions, and changes in cesarean section rates have been proposed to explain the increase in maternal mortality over the last two decades [3, 8, 10–13, 17]. In our study, maternal health factors including obesity and diabetes were associated with increasing maternal mortality, while trends in maternal age and cesarean-section rates were not in multivariable models where associations with other factors were stronger.

Maternal obesity, as measured by high body mass index, has been consistently reported to increase the risk of pregnancy complications, including thromboembolic disease, gestational diabetes mellitus, and hypertensive disorders of pregnancy [18–26]. Reports issued by state maternal mortality review panels in Virginia, Florida, and California have reported obesity as a risk factor for maternal mortality [6, 27, 28]. Elevated BMI also increases the risk of non-pregnancy-related chronic health conditions, including diabetes mellitus, identified in our study and elsewhere as a risk factor for maternal mortality [18, 21]. Chronic hypertension, cesarean section rates, and self-reported health status were also significantly associated with maternal mortality in our initial analyses, but were excluded from the final multi-level model because of the comparatively stronger associations of maternal mortality with other variables collinear with these factors. Taken together, our findings about the importance of mothers’ chronic conditions suggest additional emphasis is warranted to promote the general pre-conception health of women of childbearing age. To be sure, this endeavor is complicated by the fact that 45% of births in the U.S. are unintended [29].

Social determinants of health also play a major role in maternal health in the U.S. [30] Racial disparities are documented across multiple adverse pregnancy outcomes, with one recent study indicating that African American women were three times more likely to die as a result of pregnancy than their white peers [2]. Our analysis identified a relationship between the proportion of births to African American women and maternal mortality rates. Many factors likely play a role in perpetuating this disparity, including poor access to prenatal care and lower educational attainment, both of which were also included in our final model as independent factors [2, 31, 32]. Other socioeconomic variables significantly related to maternal mortality in initial analyses included state median income, proportion of births to women of Hispanic ethnicity, and uninsurance rates, although these were not as strong as other factors and were not retained in the most parsimonious final model. Improving the health of pregnant women will need to extend beyond addressing medical conditions to optimizing social determinants of their health as well.

The 2003 Revision of the U.S. Standard Certificate of Death introduced a checkbox to identify the pregnancy status of female decedents, changing maternal death identification. Similar changes have been reported to increase the number of maternal deaths reported, and perhaps leading to over-reporting [3, 17, 33, 34, 35]. It is possible that prior to the uniform checkbox, many pregnant decedents were not identified during completion of the death certificate, and therefore fewer maternal deaths were counted than occurred. The implementation of the new certificate likely addresses this prior ascertainment bias and therefore has increased measures of maternal mortality. Importantly, our analysis expands on previous work in terms of considering the death certificate revision in the context of other state-level factors, with 36 states adopting the new certificate by the end of our period of analysis. While adoption of the new certificate explains nearly one-third of the increase in maternal mortality rates in our multi-level model, a larger proportion is explained by other factors described above. Further, severe maternal morbidity rates have been increasing over a similar time period [36], suggesting that the increase in maternal mortality rates have a clinical etiology and do not merely reflect improvement in ascertainment.

Our analysis has certain limitations. First, we have relied on vital statistics to measure maternal mortality, which may underreport cases [34]. More active methods of surveillance exist, like those employed by the CDC’s Pregnancy Mortality Surveillance System [12, 37], though state data from that initiative are not publicly available. Levels of underreporting via vital statistics should have remained relatively constant in a given state, once controlling for the adoption of the current death certificate and employing state as a fixed effect in our models. Second, we used several national datasets as sources for state-level data. Unobserved differences between these datasets and biases inherent in each may have influenced our results. However, these datasets gave us the ability to understand how large-scale trends in health of the pregnant cohort relate to maternal mortality and enabled us to expand our work beyond the proportionally smaller number of cases of maternal death. Third, data were unavailable for some important health factors that could influence maternal mortality, including factors like maternal anemia and age younger than 18. Our work should not be interpreted outside of the age range we analyzed, and does not speak to the full range of possible causes of maternal mortality. Nonetheless, our analysis contains many of the most salient factors that increase risk for adverse maternal outcomes.

## Conclusion

This state-level analysis over nearly two decades identifies several potential opportunities to address the increasing problem of maternal mortality, including addressing health concerns of women before they become pregnant and ensuring affordable access to care for women regardless of race, education and income. While part of the increase in maternal mortality in the U.S. has likely resulted from better case ascertainment through adoption of a maternal mortality prompt in a standard death certificate across all states, health problems such as obesity and diabetes, insufficient healthcare during pregnancy, and social determinants represent identifiable risks for maternal mortality that merit direct and prompt attention in population-directed public health interventions and health policies.

## Additional file

Additional file 1: Technical Appendix. Contains a variable dictionary, results of our single predictor regression analyses, pairwise correlation of the variables listed in Table 1, and results from our final model regression analysis. (TXT 19 kb)

## Abbreviations

BMI: Body Mass Index
BRFSS: Behavioral Risk Factor Surveillance System
CDC: Centers for Disease Control and Prevention
GED: General Equivalency Diploma
ICD: International Classification of Diseases
NVSS: National Vital Statistics System
